# Materia Medica for the Use of Students

**Published:** 1865-12

**Authors:** 


MATERIA MEDICA FOR THE USE OF STUDENTS. By JOHN B. BIDDLE, M.D., Prof. of Materia Medica and General Therapeutics in the Jefferson Medical College, &c., &c. With Illustrations. Philadelphia: LINDSAY & BLAKISTON. 1865.

This is an octavo volume, of 359 pages, on good paper, fair type, and containing cuts illustrating many of the indigenous articles of the materia medica. It claims to be a simple review or summary of the materia medica, designed almost exclusively for the convenience of students, while attending their college courses of instruction. It is written in a clear, concise, and pleasing style, and is well adapted to the purpose had in view by the author.

For sale by S. C. GRIGGS & Co., Lake St., Chicago, Ill.

